# Unilateral hand force control impairments in older women

**DOI:** 10.17179/excli2022-5362

**Published:** 2022-09-22

**Authors:** Hanall Lee, Young-Min Park, Nyeonju Kang

**Affiliations:** 1 Department of Human Movement Science, Incheon National University, Incheon, South Korea; 2 Neuromechanical Rehabilitation Research Laboratory, Division of Sport Science, Incheon National University, Incheon, South Korea; 3 Division of Health and Kinesiology, Incheon National University, Incheon, South Korea; 4 Sport Science Institute & Health Promotion Center, Incheon National University, Incheon, South Korea; 5 Division of Sport Science & Sport Science Institute, Incheon National University, Incheon, South Korea

**Keywords:** unilateral motor control, force control, motor dexterity, aging, older women

## Abstract

Older women may experience deficits in sensorimotor control at their upper limb because of aging progress compromising the motor system. This study aimed to investigate whether younger and older women differ in sensorimotor capabilities assessed by unilateral force control performances at a lower targeted force level. Twenty-one older and 21 younger women performed isometric unilateral force control tasks at 10 % of maximum voluntary contraction for each hand, respectively. Purdue Pegboard Test (PPT) was used to measure unilateral hand dexterity. Five force control variables (i.e., maximal and submaximal force, force error, variability, and regularity) and PPT scores were analyzed in two-way mixed ANOVAs (Group × Hand Condition), respectively. The absolute force power was analyzed in three-way mixed ANOVA (Group × Hand Condition × Frequency Band). The findings revealed that older women produced less maximal and submaximal unilateral forces than in younger women. Greater variability, regularity, and force frequency oscillations below 4 Hz were observed in older women as compared with those in younger women. Force error in the dominant hand was greater in older women than those in younger women. Finally, older women showed lower PPT scores than younger women. These findings suggested that older women may have deficits in unilateral force control capabilities as well as motor dexterity.

## Introduction

Motor functions in upper extremities are essential for performing successful activities of daily living such as buttoning the clothes, unlocking a door or holding a cup filled with water steady state in a moving train (Hoogendam et al., 2014[31]; Kilbreath and Heard, 2005[39]; Rosenbaum, 2009[72]). The aging-related functional changes in skeletal muscles often interfere with motor actions of upper limbs which can contribute to limiting independent living (Hunter et al., 2016[33]; Seidler et al., 2010[75]). Especially, older women may frequently experience motor deficits in their upper limb because of potential abnormal muscle contractions affected by decreased motor unit firing rate and greater non-contractile muscle tissue as well as hormonal changes including estrogen deficiency compromising muscle function (Maltais et al., 2009[52]; Ross et al., 1998[71]). Various neurophysiological changes in older women potentially facilitate functional impairments of upper extremities (Bhandari et al., 2016[3]; Iolascon et al., 2015[35]; Ward et al., 2008[93]).

With aging, older adults typically experience progressive reduction of muscle strength because of degenerative change in neuromuscular level leading to deficits in sensorimotor control capabilities (Seidler et al., 2010[75]; Sosnoff and Newell, 2006[80]). Interestingly, in older women, a reduction of muscle strength patterns in upper extremities was augmented, and these motor deficits may be associated with impairments in sensorimotor processing capabilities (Maltais et al., 2009[52]; Sipilä et al., 2015[76]). In line with this finding, a previous study reported that greater decreases in maximal grip strength and increases in force error during grip and pinch force control tasks were found in older women than older men (Choi et al., 2018[11]; Ranganathan et al., 2001[66]). Older women without hormone therapy revealed a significant volumetric reduction in the frontal lobe including motor-related areas and lower neural activations in the putamen than those for older women with hormone therapy, indicating a potential relation of the estrogen level to the greater release of dopamine, a crucial neurotransmitter for executing fine motor control (Barth et al., 2015[1]; Dluzen and Horstink, 2003[18]; Gardiner et al., 2004[28]; Resnick et al., 2009[68]). These findings support a proposition that sensorimotor control capabilities may be impaired in older women because of age- and menopause-related changes in motor system.

Isometric force control performances can effectively estimate fine sensorimotor control capabilities in healthy young individuals, older adults, and patients with neurological diseases (Christou, 2011[12]; Kim et al., 2020[40]; Sosnoff and Newell, 2008[79]). Elderly groups showed deficits in isometric force control at lower targeted force levels (i.e., 10-20 % of maximal voluntary contraction: MVC) (Christou, 2011[12]; Christou and Enoka, 2011[15]; Strote et al., 2020[82]). Deterioration of motor neuron properties with increased synaptic noise in aging muscles may cause greater variability of motor unit discharge rate during submaximal force control tasks at the lower targeted force levels (Laidlaw et al., 2000[44]; Matthews, 1996[56]). Importantly, older women showed significant loss of type I and II muscle fibers as compared with younger women so that decreased motor unit recruitments may contribute to higher motor variability in controlling muscle forces at lower targeted level (Christou and Carlton, 2001[14]; Doherty et al., 1993[20]; Lang et al., 2010[47]; Messier et al., 2011[59]). Thus, examining force control performances at a lower submaximal force level may effectively and precisely assess the difference of sensorimotor control capabilities in younger and older women.

During isometric force control tasks, greater force error and variability is highly related to impaired sensorimotor integration in the motor system (Kang and Cauraugh, 2015[37]). In comparison to younger adults, older adults produced greater force error and variability with increased force oscillations below 4 Hz, suggesting that aging potentially impairs sensorimotor processing capabilities (Laidlaw et al., 2000[44]; Vaillancourt et al., 2003[86]; Vaillancourt and Newell, 2003[87]). Moreover, the nonlinear approaches to estimating the force control performances posited that lower regularity in motor outputs indicates more motor adaptability contributing to enhanced fine motor control (Sturman et al., 2005[83]; Vaillancourt and Newell, 2002[88]). Sample Entropy (SampEn), one of nonlinear assessments, can be used for assessing motor adaptability by quantifying temporal structure of variability in force signals (i.e., force regularity). Higher force regularity as indicated by lower values of SampEn denotes lower motor adaptability interfering with force control performances (Lodha et al., 2010[51]). Elderly people typically showed lower motor adaptation patterns (i.e., increased force regularity) in response to altered task demands (Hu and Newell, 2011[32]). Prior findings evidenced that older women produced less maximal strength in their dominant and non-dominant arms (Lam et al., 2016[45]; Legg et al., 2021[48]; Maltais et al., 2009[52]). To sum up, estimating force error, variability, and regularity appears to be a good model to measure changes in force control capabilities related to impaired sensorimotor function in older women.

Although the level of maximal force production was related to motor control functions at submaximal targeted force levels (Christou and Carlton, 2002[13]; Marmon et al., 2011[53]), unilateral force control capabilities, especially at submaximal force, are not fully understood in older women. Thus, the purpose of this study was to determine whether younger and older women differ in sensorimotor capability assessed by unilateral force control performances at a lower targeted force level. We administered unilateral force control tasks for dominant and non-dominant hands at 10 % of MVC (Seidler et al., 2010[75]; Strote et al., 2020[82]). Force control capabilities were assessed by measuring maximal and submaximal forces, force error, variability, regularity, and force frequency structure. In addition, we used Purdue Pegboard Test (PPT) to estimate altered unilateral hand dexterity in older women (Foy et al., 2000[25]; Ross et al., 1998[71]). Thus, we hypothesized that older women would reveal (a) lower maximal and submaximal forces, (b) greater force error, variability, regularity, and absolute power in 0 - 4 Hz frequency band, and (c) less scores in PPT across two hand conditions than younger women.

## Methods

### Participants

Twenty-one older women and 21 healthy younger women participated in this study. To determine the handedness of all participants, we used the Edinburgh Handedness Questionnaire (EHQ) (Oldfield, 1971[63]). The EHQ consists of 15 questions that indicate individual's hand preference on various activities of daily living. The value of EHQ laterality index close to 100 denotes right handedness. The values of EHQ laterality index for older women (*M*±*SD = *97.38 ± 3.75) and younger women (*M*±*SD = *95.24 ± 4.32) groups supported that all participants in this study were right handed. All participants had no musculoskeletal impairments in their upper extremities. Specific demographic information on participants is shown in Table 1(Tab. 1). We confirmed that all the participants read and signed an informed consent form and experimental protocols approved by the Incheon National University's Institutional Review Board prior to starting the test.

### Experimental procedures

**Figure 1:**
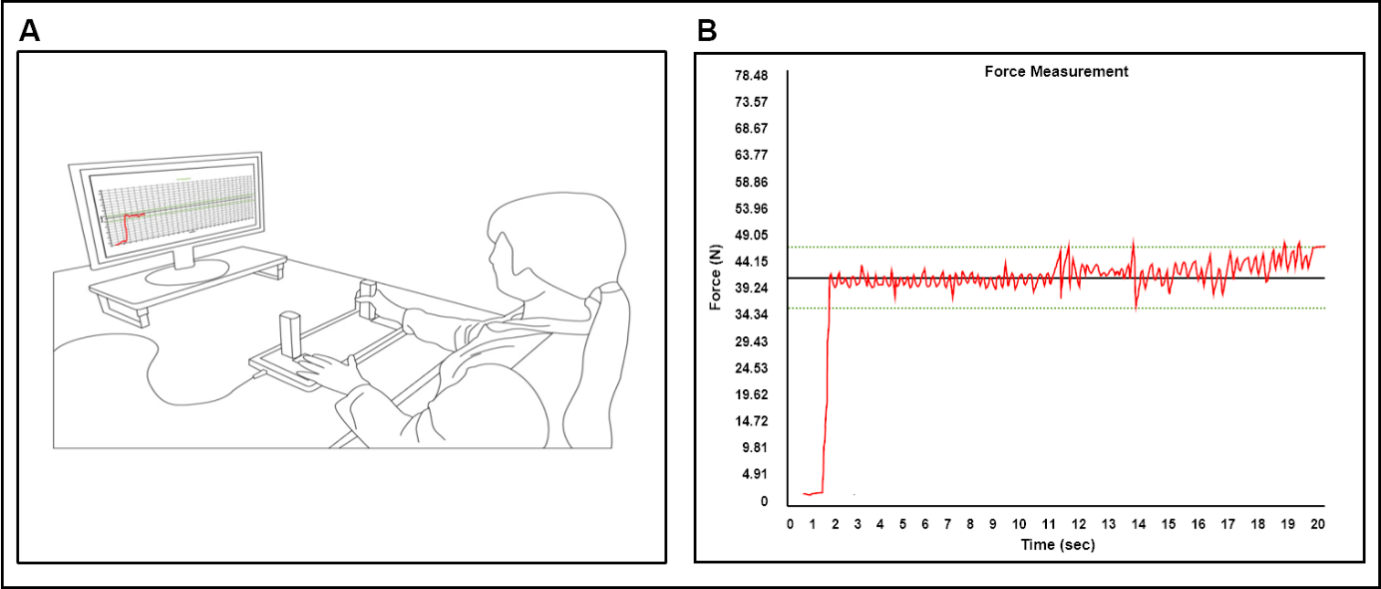
Experimental setup for unimanual force control task. (A) Participants used isometric hand-grip force measurement device to access unimanual MVC and force control capabilities at individual low targeted submaximal force level (i.e., 10 % of MVC). (B) Red line indicates individual force signals, black horizontal line denotes submaximal targeted force from individuals, and two green dotted lines are 10 % of threshold from targeted force level.

During isometric force control tasks, participants sat on a chair with upright position facing 80 cm away from a 54.6 cm LCD monitor or (1920 × 1080 pixels; 60 Hz refresh rate) and placed both arms on the table with comfortable position (elbow flexion = 20-45° and shoulder flexion = 15-20°). We used an isometric hand-grip force measurement system (SEED TECH Co., Ttd., Bucheon, South Korea) that has left and right gripping handle (a diameter = 30 mm) with fitted force transducer connected on each side (Micro Load Cell-CZL635-3135, range = 220 lbs, Phidgets Inc., Calgary, Canada). Participants were instructed to place unused hand on the pad, and both arms in a fixed position on the table to minimize unintended force caused by elbow, shoulder or trunk movements (Figure 1A(Fig. 1)).

Initially, participants completed two MVC trials (a trial duration = 5 s and rest 60 s between trials) to calculate each individual's targeted force level (i.e., 10 % of MVC). During submaximal force control tasks, participants were instructed to maintain isometric forces produced by unilateral hand (i.e., red trajectory line) to the targeted force level (i.e., white horizontal target line) for 20 s (Figure 1B(Fig. 1)). Given that participants completed 2 blocks submaximal force control tasks consisted of 3 trials for each hand respectively, six total submaximal force control trials were administered. To minimize muscle fatigues throughout the tasks, we provided 30 s of rest period between trials and 60 s of rest period between blocks. Using 16-bit analog-to-digital converter (A/D; ADS1148 16-Bit 2kSPS and a minimum detectable force = 0.0192 N) to sample all of force data at rate of 200 Hz, and INA122 amplified sampled data with voltage of 5 V (Texas Instruments Inc., Dallas, USA). We used Microsoft Visual C++ Program (Microsoft Corp., Redmond, USA) to conduct experiment procedures and utilized a customized Matlab Program (Math Works^TM^ Inc., Natick, USA) for offline analyses.

Finally, we measured hand motor dexterity function using the Perdue Pegboard (model 32020A, Lafayette Instrument Co, Lafayette, USA). During the PPT, participants were instructed to place peg tray aligned with center of upper body and insert as many pegs as possible with unilateral hand for 30 s in a vertical consecutive order. Participants completed three trials for dominant and non-dominant hand conditions, respectively (Buddenberg and Davis, 2000[5]).

### Data analyses

To minimize early initial adjustment and termination effects, we eliminated the first 3 s and last 3 s of each trial and focused on the middle 14 s of force data. Bidirectional fourth-order Butterworth filter with a cut-off frequency of 30 Hz was used for all force data. To estimate unilateral force control capabilities, we used following outcome measures: (a) maximal force (MVC) and submaximal force production (mean force), (b) force error: root-mean-square error (RMSE), (c) force variability: coefficient of variation (% CV) = standard deviation of force / mean force × 100, (d) force regularity: SampEn (see Equation 1) (Richman and Moorman, 2000[70]; Yentes et al., 2013[95]), and (e) force frequency structure. The SampEn values close to zero indicates more regular force production patterns, whereas greater values of SampEn denotes less force regularity patterns.


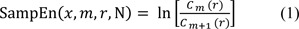


*m *stands for specific patterns of lengths, *r* is similarity criterion, and *C_m_* (*r*) represents prevalence of repetitive patterns of lengths *m* in time series only *x *itself excludes match. Consistent with the previous studies (Vaillancourt et al., 2001[90]; Yentes et al., 2013[95]), we used 2 for *m* and *r* = 0.2 × standard deviation of force signals.

To estimate force frequency structure, we quantified the absolute power across certain frequency band ranges using fast Fourier transform for power spectrum analyses. Given that the sampling rate was 200 Hz with window size 3,200, the frequency resolution was equaled to 0.0625 Hz (Fox et al., 2013[24]). Consistent with prior findings (Slifkin et al., 2000[78]; Vaillancourt et al., 2001[89]; Vaillancourt and Newell, 2003[87]), we focused on altered absolute power (N^2^) across three frequency bands: (a) 0-4 Hz; (b) 4-8 Hz; (c) 8-12 Hz. For assessing hand motor dexterity, we calculated mean Pegboard scores across three trials of each hand condition. Higher score denotes more improved hand motor dexterity.

### Statistical analyses

For statistical analyses on five force control dependent variables and Pegboard score, we used the two-way mixed ANOVAs (Group × Hand Condition; 2 × 2) with repeated measures on the last factor. Bonferroni's pair-wise comparisons were used for post hoc analysis. In addition, the absolute power from force frequency analyses was analyzed using the three-way mixed ANOVA (Group × Hand Condition × Frequency Band; 2 × 2 × 3) with repeated measures on the last two factors. For the post-hoc analysis, Bonferroni pairwise analysis was conducted. All statistical analysis procedures were performed using the IBM SPSS Statistics 25 (SPSS Inc., Chicago, IL, USA), and we set alpha level at 0.05.

## Results

### Unilateral force production: MVC and submaximal mean force

**Figure 2:**
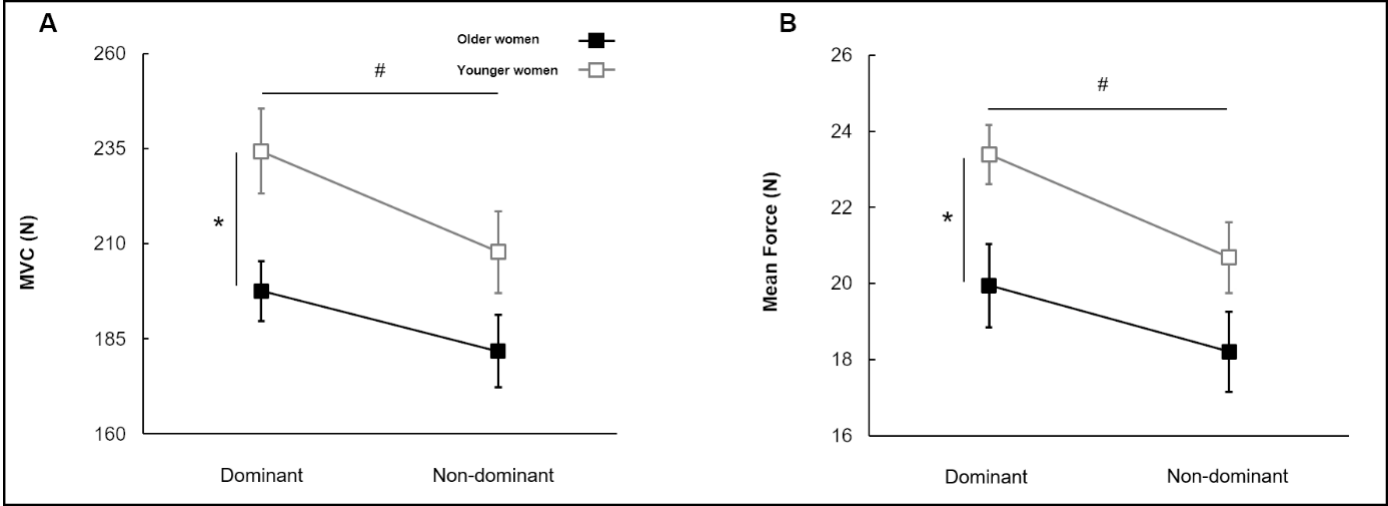
Maximum voluntary contraction and mean force for group and hand conditions (*M±SE*). (A) Maximal force (MVC) showing two main effects (i.e., Group and Hand Condition main effects). (B) Submaximal force production (mean force) showing two main effects (i.e., Group and Hand Condition main effect). *Asterisk* (*) indicates a significant difference between groups. *Number sign* (#) means a significant difference between hand conditions. (*P* < 0.05).

The two-way mixed ANOVA on the MVC revealed significant two main effects (Figure 2A(Fig. 2)): (a) group [*F*(1, 40) = 5.813; *P* = 0.021; partial η^2^ = 0.127] and (b) hand condition [*F*(1, 40) = 19.308; *P* < 0.01; partial η^2^ = 0.326]. Collapsed across group condition, MVC in non-dominant hand was significantly less than in dominant hand. Further, collapsed across hand condition, MVC in older women was significantly less than in younger women. Similarly, Group × Hand Condition (2 × 2) mixed ANOVA on the submaximal mean force showed two significant main effects (Figure 2B(Fig. 2)): (a) group [*F*(1, 40) = 5.366; *P* = 0.026; partial η^2^ = 0.118] and (b) hand condition [*F*(1, 40) = 19.308; *P* < 0.01; partial η^2^ = 0.326]. The analysis revealed that the non-dominant hands showed decreased submaximal mean force as compared to dominant hand collapsed across two groups, and older women generated significantly less submaximal mean force than younger women collapsed across two hands.

### Unilateral force control: error, variability, regularity and frequency structure

**Figure 3:**
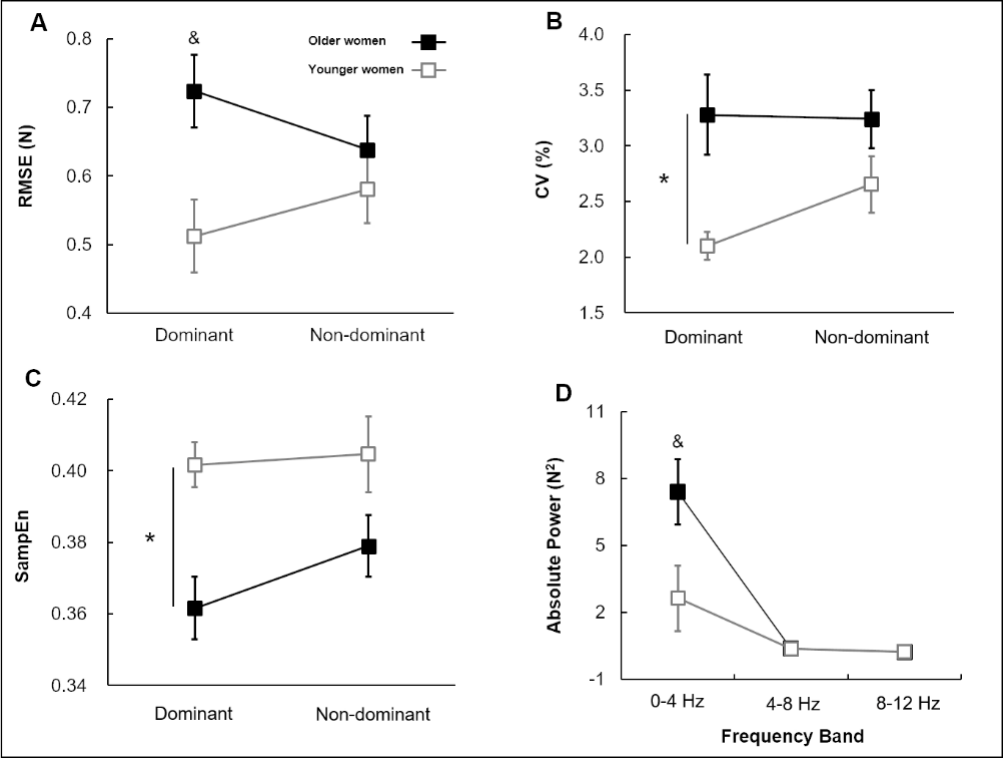
Force control for group and hand conditions (*M±SE*). (A) Force error (RMSE) showing a significant Group × Hand Condition interaction. (B) Force variability (CV) showing Group main effect. (C) Force regularity (SampEn) showing Group main effect. (D) Absolute power indicating a significant Group × Hand Condition × Frequency Band interaction. *Asterisk* (*) indicates a significant difference between groups. *Ampersand *(&) signs denote a significant difference between hand conditions. (*P* < 0.05).

The two-way mixed ANOVA on the RMSE showed a significant Group × Hand Condition interaction [*F*(1, 40) = 4.368; *P* = 0.043; partial η^2^ = 0.098; Figure 3A(Fig. 3)]. Post-hoc analysis revealed that older women showed higher values of RMSE in dominant hand than younger women (*P* = 0.007). The analysis on the CV revealed a significant group main effects [*F*(1, 40) = 7.068; *P* = 0.011; partial η^2^ = 0.150; Figure 3B(Fig. 3)]. The analysis showed that older women produced higher values of CV than younger women who collapsed across hand conditions. Group × Hand Condition mixed ANOVA on the SampEn demonstrated a significant group main effect [*F*(1, 40) = 9.561; *P* = 0.004; partial η^2^ = 0.193; Figure 3C(Fig. 3)]. Collapsed across hand conditions, the values of the SampEn in older women were significantly lower than in younger women. The Group × Hand Condition × Frequency Band mixed ANOVA on the absolute power revealed a significant three-way interaction [*F*(1, 40) = 5.261; *P* = 0.027; partial η^2^ = 0.116; Figure 3D(Fig. 3)]. The post-hoc analysis showed that the absolute power values at 0-4 Hz in dominant hand of older women were significantly higher than in younger women.

### Motor dexterity function: Perdue Pegboard Test

The Group × Hand condition mixed ANOVA on the PPT score showed two significant main effects: (a) group [*F*(1, 40) = 21.751; *P* < 0.001; partial η^2^ = 0.352] and (b) hand condition [*F*(1, 40) = 30.601; *P* < 0.001; partial η^2^ = 0.433]. The analysis showed that older women had lower PPT scores (*M*±*SE* = 13.341±0.319) than younger women (*M*±*SE* = 15.444±0.319) across hand condition. Further, collapsed across two groups, PPT score in dominant hand (*M*±*SE* = 14.817±0.235) was significantly higher than in non-dominant hand (*M*±*SE* = 13.968 ± 0.241). 

## Discussion

To our knowledge, no research has investigated unilateral force control capabilities in older women especially at submaximal force. This study examined potentially different sensorimotor functions between older and younger women by focusing on unilateral force control performances at 10 % of MVC. Specifically, older women produced less maximal and submaximal force with their unilateral dominant and non-dominant hands than younger women. Unilateral force control capabilities in older women were significantly impaired as indicated by greater force error, variability, regularity, and force frequency oscillations in comparison to those in younger women. Additionally, older women showed more deficits in unilateral motor dexterity functions than younger women.

Consistent with the hypothesis in the present study, previous research reported that older women showed lower maximal and submaximal force production across their dominant and non-dominant hands (Li et al., 2018[49]; Soyupek et al., 2006[81]; Suuronen et al., 2016[85]). Older women constantly showed a reduction of maximal grip strength after age 45 (Frederiksen et al., 2002[26]). Prior findings suggested that impaired muscle strength in older adults may be associated with decreasing muscle mass influenced by a reduced number of muscle fibers (i.e., type II muscle fiber) (Roos et al., 1997[71]; Vandervoort, 2002[91]). Progressive degenerative changes in the neuromuscular system normally appear in individuals with age 50 to 60 years (Doherty, 2003[19]; Samson et al., 2000[74]). Further, neuromuscular degenerative change induces atrophy of type II muscle fibers and re-innervates with surviving type I motor units resulting in decreased strength in older individuals (Faulkner et al., 2007[23]; Maltais et al., 2009[52]). In fact, older women showed lower muscle mass and isometric strength at the elbow than younger women (Landers et al., 2001[46]). Taken together, these findings indicated that older women may experience muscle weakness in their upper extremities partially due to degenerative change in neuromuscular properties (Doherty, 2003[19]; Surakka et al., 2003[84]).

The present study revealed that, at 10 % of MVC, older women showed deficits in force control capabilities including greater force error and force variability. Previous studies found that controlling forces at the lower targeted levels (e.g., 2-20 % of MVC) require more challenging and complex neuromuscular functions than those at the higher force levels (Enoka et al., 2003[22]; Galganski et al., 1993[27]; Keogh et al., 2006[38]). Further, different force control capabilities between older and young adult groups significantly increased at 10 % of MVC as elderly people exhibited more force errors and variability during isometric force control tasks (Burnett et al., 2000[6]; Christou and Tracy, 2005[16]; Christou and Enoka, 2011[15]; Laidlaw et al., 2000[44]). The current findings are expanding the prior results to the older women by showing more force control impairments at the lower targeted force level. Typically, elderly people showed impaired force control capabilities presumably related to progressive impairments in motor unit properties as a result of aging process (Enoka et al., 2003[22]; Kornatz et al., 2005[42]). The neuromuscular system in older adults may induce compensatory changes to apotheosis spinal motor neurons by spreading additional axonal sprouts for functional linkage to disconnected muscle fibers leading to a single motor unit with higher density of muscle fibers (Kanda and Hashizume, 1989[36]; Piasecki et al., 2016[65]). Consequently, the threshold of discharging action potential may decrease for re-innervated motor units at the lower targeted force level resulting in higher force fluctuations in elderly people as compared with younger adults (Galganski et al., 1993[27]; Piasecki et al., 2016[65]). Higher variability in action potential discharge rate of motor neurons may compromise an ability to perform accurate movements in older women at the lower targeted force level (Harris and Wolpert, 1998[30]; Martin et al., 2015[55]).

Higher force regularity at 10 % of MVC in older women supports the hypothesis of the impaired complexity as a part of aging process (Rey-Robert et al., 2011[69]; Vaillancourt and Newell, 2002[88]; Vieluf et al., 2015[92]). The assumption posited that the regularity of force output patterns may generate from the contraction patterns of muscle fibers potentially affected by interaction of neuronal and hormonal variables in the complexity system (Pette and Staron, 2000[64]). Impairments in muscle components (e.g. losing α-motor neurons and hormone receptors, and denervated muscle fibers) are a consequence of progressive aging, and these structural changes may lead to less adaptive motor control behaviors (e.g., more regular force production patterns) in older adults (Vaillancourt and Newell, 2002[88]). Previous findings have shown that older adults have more regular force output patterns during force control (Chen et al., 2017[8]; Vieluf et al., 2015[92]). Thus, our findings supported an idea that potential effects of losing structural integrity in the neuromuscular system on more stereotyped motor behaviors may be additionally observed in older women.

Greater absolute power in the 0-4 Hz frequency band provides an additional deficit in sensorimotor processing in older women (Slifkin et al., 2000[78]; Vaillancourt et al., 2001[89]). Aging may interfere with sensorimotor integration functions, and further greater force oscillation patterns associated with impaired sensorimotor processing significantly appeared in the lower frequency band (< 4 Hz) (Fox et al., 2013[24]; Vaillancourt and Newell, 2003[87]). Lodha and Christou posited that low frequency oscillation may originate from the communication between brain and motor neuron by descending drive of neural signals (Fox et al., 2013[24]; Lodha and Christou, 2017[50]; Moon et al., 2014[60]). Moreover, greater force variability in older adults may be associated with slow sensorimotor process below 4 Hz of the force oscillations (Vaillancourt et al., 2003[86]; Vaillancourt and Newell, 2003[87]; Wessberg and Vallbo, 1996[94]). Increased absolute power at 0-4 Hz in older women may be related to greater synaptic noise that potentially induced greater variability of motor neuron pool activations partially related to aging process of degenerative change in motor neurons (Laidlaw et al., 2000[44]; Matthews, 1996[56]; Moritz et al., 2005[61]). These findings suggested that degenerative neuromuscular change as potential contributor for the impaired sensorimotor process in older women may induce high force amplitude in the frequency band between 0 to 12 Hz.

Not surprisingly, in the present study, older women revealed more impaired unilateral motor dexterity at dominant hand with lower PPT scores than younger women. These findings are consistent with previous findings that aging causes impairments in hand function in older women (Edmonds, 2003[21]; Ranganathan et al., 2001[66]). Older women showed lesser PPT scores than younger women on dominant and non-dominant hand respectively (Ranganathan et al., 2001[66]; Soyupek et al., 2006[81]). Older women who experienced symptoms of hormonal deficiency and aging progress may facilitate deficits in motor dexterity and processing speed of cognitive function (Greendale et al., 2009[29]; McEwen, 2001[58], 2002[57]). Taken together, age-related deficits in motor control and drastic hormonal change may impair dexterity at dominant hand in older women.

Older women often experience menopausal symptoms that may result in impairments in motor function with dramatic changes in hormonal levels (Maltais et al., 2009[52]). Lower sex hormone levels (i.e., estrogen and progesterone) may be associated with degenerative change in neuromuscular system (Cheng et al., 2009[9]; Kurina et al., 2004[43]; Rathnayake et al., 2021[67]) and neurotransmitter system (Barth et al., 2015[1]) leading to impaired muscle strength and movement control. Prior studies suggested a possibility that the level of estrogen may affect muscle strength (Chidi-Ogbolu and Baar, 2018[10]; Ikeda et al., 2019[34]). Sitnick and colleagues posited that estrogen could stimulate insulin like growth factor 1 (IGF-1) receptors in skeletal muscles contributing to muscle protein synthesis (Sitnick et al., 2006[77]). Decreased estrogen level and IGF-1 receptors may impair the neuromuscular system that potentially induce decreased strength in older women (Brown, 2008[4]). Compared with premenopausal women, the symptoms of muscle weakness such as lower pinch and grip forces in upper limbs were observed in postmenopausal women (Kurina et al., 2004[43]; Rathnayake et al., 2021[67]). For the degenerative change in neurotransmitter systems, lower level of sex hormone may interfere with gamma-aminobutyric acid (GABA) and dopamine transmission efficiency (Barth et al., 2015[1]; Deligiannidis et al., 2013[17]; Marshall, 2008[54]). GABA is a predominant inhibitory neurotransmitter in the human central nerve system contributing to successful movement control so that deficits in the GABA system may compromise sensorimotor processing (Cassady et al., 2019[7]; Marshall, 2008[54]). Moreover, estrogen deficiency may impair the dopamine transmission efficiency, potentially associated with deterioration in accurate and adaptive motor control capabilities (Barth et al., 2015[1]; Klein et al., 2019[41]; Vaillancourt and Newell, 2002[88]). Estradiol-treated postmenopausal women showed enhanced sensorimotor performances during standing balance and finger tapping task with advanced motor adaptability than the non-estradiol-treated women (Bayer and Hausmann, 2010[2]; Gardiner et al., 2004[28]; Naessen et al., 2007[62]). These cumulative findings raise a possibility that unilateral motor impairments in upper extremities (i.e., decreased strength, impaired sensorimotor control, and stereotyped force production patterns) in older women may be influenced by menopause symptoms combined with aging effects.

Despite impairments in unilateral force control and dexterity in the older women, these findings should be carefully interpreted. As we discussed above, reduced grip strength and compromised force control capabilities in older women may be related to degenerative neuromuscular change in aging process as well as motor symptoms of sex hormone deficiency (Barth et al., 2015[1]; Hunter et al., 2016[33]; Maltais et al., 2009[52]). Nevertheless, the actual hormone levels for both older and younger women were not quantified. Whether potential menopause symptoms related to neurotransmitter system influence on unilateral force control and motor dexterity need to be further investigated. Future studies should examine the effects of specific estrogen levels and neurotransmitter facilitation (e.g., GABA, dopamine levels, and integrity of neurotransmitter receptors) on unilateral motor performances to provide more comprehensive information on the impaired neuromuscular system. 

## Conclusion

The current study identified that older women had impairments in unilateral motor control as compared with younger women. Specifically, older women produced lower hand-grip forces, and further revealed impairments in force control capabilities at the lower targeted force as indicated by greater force error, variability, regularity, and frequency oscillation below 4 Hz. In addition, older women showed lower unilateral motor dexterity (i.e., lower PPT scores) than younger women. These findings suggested that older women may experience impairments in unilateral force and movement control capabilities at upper limbs partially due to age-related deficits in the neuromuscular system. Future studies should investigate potential rehabilitation programs to facilitate functional improvements in the upper extremities for older women.

## Declaration

### Conflict of interest 

The authors declare that they have no competing interests.

### Funding 

This work was supported by the Incheon National University Research Grant (2022-0094) to NK.

### Author contributions

Hanall Lee: Conceptualization, visualization, methodology, data analysis, writing - original draft, review & editing. Young-Min Park: Supervision, visualization, writing - review & editing. Nyeonju Kang: Conceptualization, methodology, funding acquisition, project administration, software, supervision, validation, visualization, writing - review & editing.

### Availability of data and material

All relevant data are within the manuscript.

## Figures and Tables

**Table 1:**
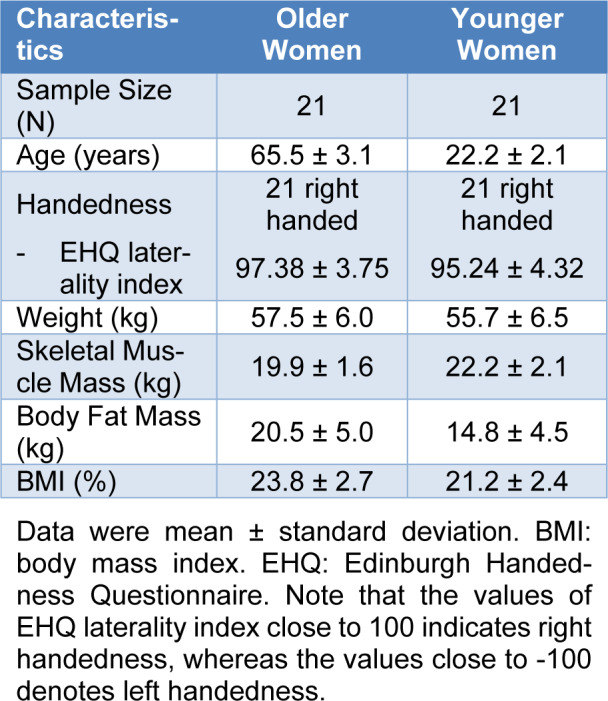
Demographics of the participants
